# Addressing the Role of Smart Robotic Health Assistants Within the Human-Machine Frontier of Geriatric Healthcare

**DOI:** 10.1093/geroni/igaa057.1319

**Published:** 2020-12-16

**Authors:** Nadia Firdausya, Alex Bishop, Barbara Carlson, Weihua Sheng

**Affiliations:** 1 Oklahoma State University, stillwater, Oklahoma, United States; 2 Oklahoma State University, Stillwater, Oklahoma, United States; 3 University of Oklahoma Health Sciences Center, Oklahoma City, Oklahoma, United States

## Abstract

Data for this study was acquired from three separate stakeholder focus group sessions involving nurse case managers (n = 5), social agency caseworkers (n = 5), and rural outreach providers (n = 5). Participants across all groups were asked to address the question: “When it comes to your work, what would you want a smart robot assistant to do for you?” Data from the three sessions were combined, transcribed verbatim, coded, and analyzed for thematic content. Three shared themes emerged, including health monitoring, behavioral intervention, and healthcare literacy. Relative to health monitoring, participants desired a robot that possessed functions in the form of “taking vital signs,” and “tracking water and food intake.” There was also a thematic agreement regarding behavioral intervention capabilities. Most notably, advisory stakeholders acknowledged a need for a smart robotic assistant to provide geriatric care recipients with “an alert or reminder to take medication.” This was viewed as an essential intervention for improving medication adherence. Healthcare literacy emerged as a final theme among advisory groups. In particular, participants noted that a smart robot should assist with bi-directional communication and translation of health care information and instructions as a way to “minimize impediments of care due to language barriers.” Findings will be further used to highlight how future integration of robotic health assistants represents a viable solution in helping geriatric healthcare workers work effectively alongside machines to meet the diverse care needs of older adults in both urban and rural settings.

